# Systemic Right Ventricle and Myocardial Bridging: Hemodynamics and Surgical Solutions in Adult Congenital Heart Disease

**DOI:** 10.1016/j.jaccas.2026.109449

**Published:** 2026-08-26

**Authors:** Antonio Carlos Escorel de Almeida Neto, Luiz Rafael Pereira Cavalcanti

**Affiliations:** Department of Cardiovascular Surgery, Instituto de Medicina Integral Prof. Fernando Figueira, Recife, Pernambuco, Brazil

**Keywords:** congenital heart defect, myocardial ischemia, transposition of the great arteries

**Figure:**
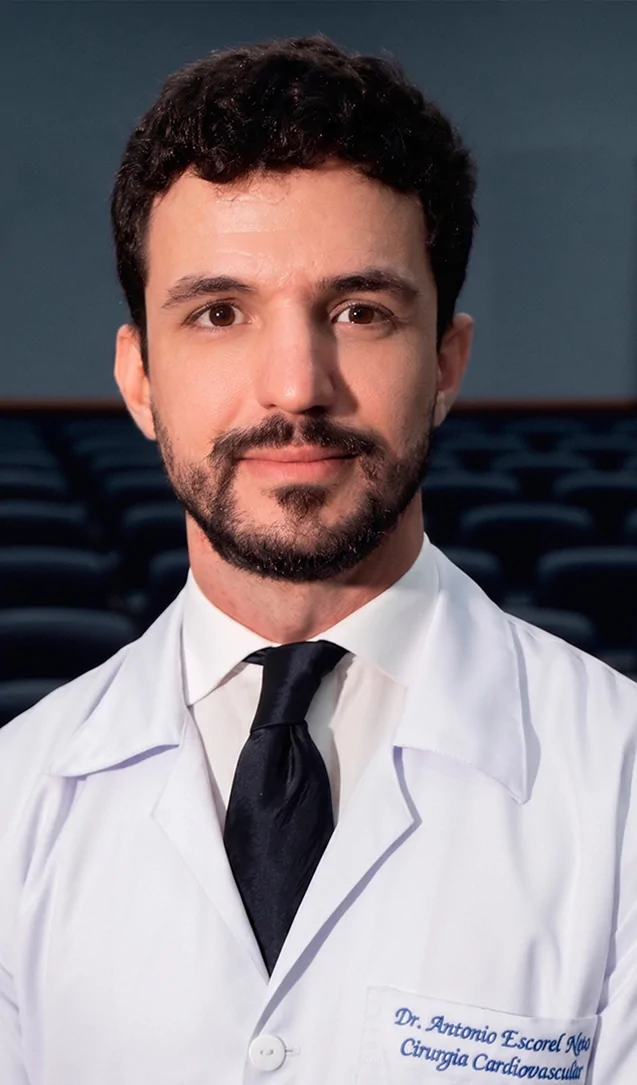

D-transposition of the great arteries is a congenital anomaly characterized by atrioventricular concordance and ventriculoarterial discordance, creating parallel pulmonary and systemic circulations. Without intervention, this condition is incompatible with survival. Before the widespread adoption of the arterial switch operation, the Senning^1^ and Mustard^2^ procedures were the standard of care. These operations redirect venous return at the atrial level, allowing the morphologic right ventricle (RV) to support the systemic circulation. This surgical strategy transformed the prognosis of D-transposition of the great arteries, enabling a cohort of patients to reach adulthood. However, the systemic RV must sustain systemic pressures, predisposing it to progressive hypertrophy, dysfunction, and ischemia over time.^3^

In this issue of *JACC: Case Reports*, Yoshida et al^4^ present a case of a 21-year-old man with exertional ischemia 2 decades after a Senning procedure and a left subclavian flap aortoplasty. Multimodality imaging identified ascending aortic stenosis and a myocardial bridge (MB) over the left anterior descending (LAD) artery. This case demonstrates a pathophysiological cascade: a congenital coronary anomaly, which is often asymptomatic in structurally normal hearts, became hemodynamically restrictive secondary to increased afterload and systemic RV hypertrophy.

Myocardial bridging is a common anatomical variant that rarely requires surgical intervention.^5^ However, the altered hemodynamics of a systemic RV modify this interaction. Because the RV hypertrophies to overcome systemic vascular resistance—exacerbated in this patient by ascending aortic stenosis—the extravascular compressive forces exerted on the tunneled LAD increase. This mechanism creates a cycle of supply-demand mismatch during systole and impaired diastolic relaxation, leading to the angiographic “milking” effect and documented RV ischemia.

The standard surgical treatment for symptomatic MB is supra-arterial myotomy. This technique is effective and often represents the first-line approach in pediatric or young adult populations before irreversible endothelial dysfunction or atherosclerosis develops in the proximal segment.^6^ The morphologic criteria of the MB, however, determine the feasibility of this approach. The bridge in this case was deep (9.5 mm) and long (34 mm). Executing a deep myotomy in a pressurized, hypertrophied systemic RV introduces a risk of ventricular wall perforation or subsequent ventricular aneurysm formation.^7^ Consequently, coronary artery bypass grafting emerged as the indicated surgical option.

The graft selection detailed by the authors highlights the challenges of reoperations in congenital heart surgery. Arterial grafting is the standard for LAD revascularization, but the left internal mammary artery was unavailable because of a neonatal left subclavian flap aortoplasty. The authors used a free right internal mammary artery. To mitigate the risk of ostial stenosis associated with anastomosing an arterial conduit directly to a Dacron graft in ascending aorta, a short segment of saphenous vein graft was interposed. This composite configuration maximizes graft patency and adapts to the patient's prior surgical anatomy.

Yoshida et al^4^ provide a reminder that late-onset ischemia in the adult congenital heart disease population requires structural and functional investigation. Systemic RV ischemia cannot be assumed to be solely microvascular or a definitive consequence of ventricular mass. Superimposed anatomical lesions, such as myocardial bridging or epicardial coronary disease, must be actively evaluated using imaging modalities such as computed tomography angiography and stress positron emission tomography. When identified, a tailored surgical approach that respects the modified physiology and previous surgical interventions can restore myocardial perfusion and preserve ventricular function.

## Funding Support and Author Disclosures

The authors have reported that they have no relationships relevant to the contents of this paper to disclose.
